# Perceptual assimilation of lexical tone: The roles of language experience and visual information

**DOI:** 10.3758/s13414-014-0791-3

**Published:** 2014-12-03

**Authors:** Amanda Reid, Denis Burnham, Benjawan Kasisopa, Ronan Reilly, Virginie Attina, Nan Xu Rattanasone, Catherine T. Best

**Affiliations:** 1 MARCS Institute, University of Western Sydney, Locked Bag 1797, Penrith, NSW 2751 Australia; 2 Department of Computer Science, National University of Ireland, Maynooth, Ireland; 3 Department of Linguistics, Macquarie University, Sydney, NSW 2109 Australia

**Keywords:** Lexical tones, Nonnative speech perception, Perceptual assimilation model, Auditory–visual speech

## Abstract

Using Best’s (1995) perceptual assimilation model (PAM), we investigated auditory–visual (AV), auditory-only (AO), and visual-only (VO) perception of Thai tones. Mandarin and Cantonese (tone-language) speakers were asked to categorize Thai tones according to their own native tone categories, and Australian English (non-tone-language) speakers to categorize Thai tones into their native intonation categories—for instance, question or statement. As comparisons, Thai participants completed a straightforward identification task, and another Australian English group identified the Thai tones using simple symbols. All of the groups also completed an AX discrimination task. Both the Mandarin and Cantonese groups categorized AO and AV Thai falling tones as their native level tones, and Thai rising tones as their native rising tones, although the Mandarin participants found it easier to categorize Thai level tones than did the Cantonese participants. VO information led to very poor categorization for all groups, and AO and AV information also led to very poor categorizations for the English intonation categorization group. PAM’s predictions regarding tone discriminability based on these category assimilation patterns were borne out for the Mandarin group’s AO and AV discriminations, providing support for the applicability of the PAM to lexical tones. For the Cantonese group, however, PAM was unable to account for one specific discrimination pattern—namely, their relatively good performance on the Thai high–rising contrast in the auditory conditions—and no predictions could be derived for the English groups. A full account of tone assimilation will likely need to incorporate considerations of phonetic, and even acoustic, similarity and overlap between nonnative and native tone categories.

## Introduction

In second language learning, the relationship between the phonological and phonetic properties of the first (L1) and the second (L2) languages influences L2 perception and understanding (e.g., Best, 1995; Kuhl, 1991, 1992). One of the most influential and successful models of this process is the *perceptual assimilation model* (PAM; Best, 1995). Most applications of PAM to L2 perception concern the auditory perception of consonants and vowels, but here we applied the PAM to the auditory–visual perception of lexical tones. Native adult speakers of three tone languages (Thai, Cantonese, and Mandarin) and of a nontone language (English) were tested for their cross-language category assimilations of the five Thai tones, and these data were used, via PAM procedures, to predict the discrimination of Thai lexical tones. To provide context for the study, explication of the PAM is presented first, followed by a review of the relevant literatures on lexical tone perception and visual speech perception.

### Perceptual assimilation model and cross-language mapping

A listener’s background with linguistically relevant native (L1) speech segments, or *phonemes*, exerts an influence on the perception of nonnative (L2) speech segments, or *phones*. The degree of success in perceiving L2 phones has been modeled most frequently by the PAM (Best, 1995; Best & Tyler, 2007), the speech learning model (SLM; Flege, 1995, 2002), and the native-language magnet model (NLM; Kuhl, 1991, 1992). Here we examine lexical tone in the context of the PAM, which seeks to explain the perception of unknown L2 phones by naïve perceivers, rather than the SLM, which is more concerned with L2 learning, or the NLM, which focuses on discrimination among tokens falling within single L1 categories. The PAM framework, traditionally applied to segments (consonants and vowels), and especially to minimal contrasts of segments, proposes that nonnative phones tend to be perceived according to their degree of similarity to native segments or segment combinations that are close to them in the native phonemic space. The perceived phonetic distance between each of two contrasting L2 phones and the closest L1 segment(s) (if any) is proposed to lead to differences in L2 contrast discriminability.

The PAM outlines a number of patterns of perceptual assimilation. For example, the Ethiopian Tigrinya ejectives [p’] and [t’] are perceived by English language environment listeners to be most similar to English /p/ and /t/ ([p^h^] and [t^h^]), respectively, so a two-category (TC) assimilation pattern applies, resulting in very good discrimination (Best, McRoberts, & Goodell, 2001). In the category goodness difference (CG) pattern, two nonnative phones are assimilated into the same native category but differ in their degrees of discrepancy from the native “ideal.” This CG pattern is the case with the Zulu voiceless versus ejective velar stops, [k^h^]–[k’], which are perceived by English listeners as being a good versus a noticeably deviant English /k/ ([k^h^]) (Best et al., 2001); the greater the difference in category goodness, the better the predicted discrimination performance. In the single-category (SC) pattern, two nonnative phones are assimilated into the same native category, with poor discrimination being predicted (Best, 1995) and observed, as for the Zulu plosive versus implosive [b]–[ɓ], both of which English listeners assimilate to their native /b/ (Best et al., 2001). Specifically, the predicted order of discriminability associated with these three assimilation patterns, which we will refer to as *cross-language mappings* in this article, would be TC > CG > SC (Best, 1995; Best et al., 2001).

If one phone of a pair is not consistently identified as falling within any single native category, whereas the other is, they are classed as *uncategorized* versus *categorized* (UC; good discrimination predicted), as has been observed for Japanese L2-English learners’ assimilation of the Australian English vowels [3]–[ʉ:] (the vowels in NURSE and GOOSE, respectively) to Japanese /u , ua, au, e:/ (uncategorized: assimilations were split across these L1 vowels) versus /u:/ (categorized), respectively (Bundgaard-Nielsen, Best, Kroos, & Tyler 2011a; Bundgaard-Nielsen, Best, & Tyler 2011b). If both phones are not consistently identified as falling within any single native category, they are both uncategorizable (UU; with discrimination performance being dependent on the perceived magnitude or salience of the phonetic difference). In the nonassimilable (NA) pattern, nonnative phones fail to be assimilated to the native speech system, but are instead perceived as nonspeech sounds. NA contrasts can be discriminated by attending to acoustic differences, with discriminability depending on their psychoacoustic similarity. For example, in the case of Zulu click consonant contrasts, English listeners cannot assimilate either phone into their native phoneme space; they report that the sounds do not sound like speech, but instead like nonspeech events such as a twig snapping or a cork popping, and they discriminate them quite well (Best, Levitt, & McRoberts, 1991).

Empirical assessment of perceived phonetic similarity is afforded by cross-language category assimilation experiments in which L2 phones are classified as instances of particular phoneme categories in the perceiver’s L1, then rated for goodness of fit to the L1 category. Using this method, it has been shown that such cross-language mapping patterns (henceforth referred to as *cross-mapping patterns*) can predict L2 consonant and vowel discrimination accuracy (Best, Faber, & Levitt 1996; Guion, Flege, Akahane-Yamada, & Pruitt, 2000; Polka, 1995). However, some authors have pointed out potential problems with PAM: Harnsberger (2001), in an investigation of consonant discrimination patterns, found a large proportion of uncategorizable assimilations, and furthermore, although the mean discrimination scores supported the PAM predictions, there was an unexpected range of discrimination scores for single-category and category goodness assimilations. This underlines the need for more research on the utility of the PAM; here, we explored whether category assimilation patterns can predict the L2 discrimination accuracy of lexical tones.

### Lexical tone perception and perceptual assimilation

**Fig. 1:**
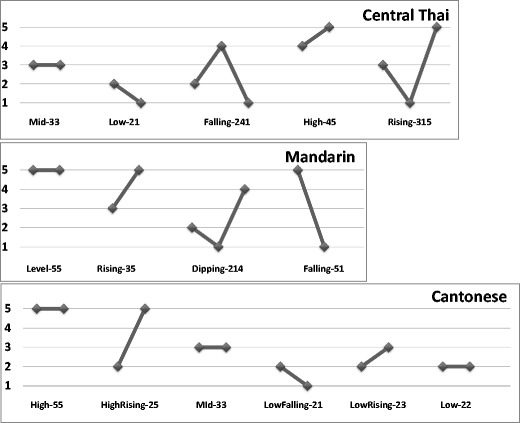
Schematics of the tone types (on one syllable), charted by Chao (1930) values, in the three target tone languages: Thai, Mandarin, and Cantonese (Bauer & Benedict, 1997). A tone is labeled using integers from 1 to 5, with 5 representing the *highest* pitch and 1 the *lowest*. Two integers are used to indicate the onset and offset pitch height of the tone, with a third, middle integer being added for contour tones at the point of inflection. Note that Cantonese 25 is also described as 35 (i.e., both are allotones of the same lexical tone), and Cantonese 55 is also described as 53 (see L. K. H. So, 1996). The tones are ordered according to traditional convention—that is, 0–4 for Thai, 1–4 for Mandarin, and 1–6 for Cantonese

Tone languages comprise about 70 % of the world’s languages (Yip, 2002), and in these, the fundamental frequency (F0) height and contour parameters change the meanings of words. In Thai, there are three relatively static or *level* tones—for example, [k^h^á:], high tone, meaning “to trade”; [k^h^ā:], mid tone, “to be stuck”; and [k^h^à:], low tone, “galangal, a Thai spice”—and two dynamic or *contour* tones—for example, [k^h^ǎ:], rising tone, meaning “leg,” and [k^h^â:], falling tone, “to kill.” Tone languages vary in their tone systems; for example, Cantonese has three level and three contour tones, and Mandarin has one level and three contour tones. Figure 1 shows the tone types in Thai, Cantonese, and Mandarin using the tone notation system proposed by Chao (1930).

Experience with a particular tone language influences listeners’ auditory identification and discrimination of nonnative tones (Burnham et al., 2014b; Lee, Vakoch, & Wurm, 1996; Qin & Mok 2011; So & Best, 2010, 2011, 2014; Wayland & Guion, 2004). In general, tone experience in one language facilitates that in another tone language, but in some cases, inhibition is due to specific tone-language experience. For example, So and Best (2010) found that Cantonese listeners incorrectly identified Mandarin falling (51) tones as level (55), and rising (35) tones as dipping (214), significantly more often than Japanese and English listeners made these errors; it also appears that the perception of level tones in a foreign tone language may be directly (Qin & Mok, 2011) or inversely (Chiao, Kabak, & Braun, 2011) affected by the number of level tones in the listener’s native tone language.

In a category assimilation study investigating the extension of PAM to the perception of tones, So and Best (2014) showed that Cantonese listeners were able to categorize three out of the four Mandarin tones into a Cantonese tone category: A Mandarin level (55) was assimilated to a Cantonese high (55) tone, a Mandarin falling (51) to a Cantonese high (55) tone, and a Mandarin rising (35) to a Cantonese high-rising (25) tone, but a Mandarin dipping (214) tone was not clearly assimilated to any one category. Predictions of discrimination performance from these cross-mapping data generally supported the PAM predictions (two-category [TC] pairs were discriminated better than category goodness [CG] pairs), although they found large differences in performance among the UC pairs. So and Best (2010) reported that Japanese pitch-accent speakers were also able to categorize Mandarin tones into their native pitch-accent categories, choosing the pitch-accent categories that were phonetically similar to the Mandarin tones. However, like Cantonese listeners, they also had difficulty categorizing the Mandarin dipping (214) tone, and once again, they showed large discrepancies in the UC pairs in the discrimination data.

Applying the PAM to English listeners’ L2 lexical tone perception is complicated. Given that French (also a nontone language) speakers were found to perceive tones in a more psychophysical manner—that is, less categorically—than Mandarin speakers, Hallé, Chang, and Best (2004) suggested that tones could be perceived by non-tone-language speakers as either uncategorized (UU) speech categories or as nonspeech or musical melodies (NA). Alternatively, there is evidence that tones could be assimilated to specific intonation (prosodic) categories in nontone languages (So & Best 2008, 2011, 2014), although it is quite possible that native intonation categories might not have the same degree of influence as native tone categories (Hallé et al., 2004). Individual single Mandarin tone words were generally categorized by native English speakers as follows: level (55) as a flat pitch, rising (35) as a question, dipping (214) as uncertainty or a question, and falling (51) as a statement (So & Best 2008, 2014). When tones were presented in sentence form to English and French speakers, and using slightly different categories, level (55) and dipping (214) tones were perceived predominantly as statements, rising (35) tones as questions, and falling (51) tones as statements for English speakers but as exclamations for French speakers (So & Best, 2011, 2014). English performance was analogous to that of the Japanese listeners in So and Best (2010), but their results provide more specific insight into the categorization and discrimination relationship; English listeners (So & Best, 2014) showed large discrepancies among the UC pairs, with one UC pair being associated with much better discrimination than the TC pairs. Although So and Best (2014) concluded that PAM principles were generally upheld for the perception of nonnative tones, some aspects of the findings were less obviously related to PAM principles. Thus, more data are required, especially using different target and listener tone languages.

### Visual cues for lexical tone and perceptual assimilation

None of the studies above examined visual (lip, face, head, and neck motion) influences on category assimilation. Visual speech information is known to benefit speech perception, both under difficult listening conditions (e.g., Sumby & Pollack, 1954) and in completely undegraded (McGurk & McDonald, 1976) conditions (see Campbell, Dodd, & Burnham, 1998, and Bailly, Perrier, & Vatikiotis-Bateson, 2012, for a comprehensive collection of articles). In addition, in the last decade it has been established that there is visual information for tones. Burnham, Ciocca, and Stokes (2001a) asked native speaking Cantonese participants to identify Cantonese words in auditory–visual (AV), auditory-only (AO), and visual-only (VO) modes. Performance was equivalent in the AO and AV modes. However, in the VO condition, tones were identified slightly but statistically significantly above chance levels under certain conditions—in running speech (as opposed to isolated words), on monophthongal vowels (as opposed to diphthongs), and on contour (as opposed to level) tones. Auditory–visual augmentation for the identification of tones has also been found for speech in noise for both Mandarin (Mixdorff, Charnvivit, & Burnham 2005a) and Thai (Mixdorff, Hu, & Burnham 2005b) listeners. Such visual information for tone is also available to and used by non-tone-language speakers to discriminate lexical tones (Burnham, Lau, Tam, & Schoknecht 2001b; Burnham et al., 2014b; Burnham, Vatikiotis-Bateson, Yehia, Ciocca, Haszard Morris, Hill, and Reid, 2014c; Smith & Burnham, 2012), and Smith and Burnham found that tone-naïve listeners outperformed native listeners in VO tone discrimination, additionally suggesting that the visual information for tone may be underused by normal-hearing tone-language perceivers.

Although perceivers clearly use visual information to distinguish tones, it is unclear what specific cues are used. Nevertheless, there are some preliminary indications. Chen and Massaro (2008) observed that Mandarin tone information was apparent in neck and head movements; and subsequent training drawing attention to these features successfully improved Mandarin perceivers’ VO identification of tone. In that study, participants were given very general information—for example, Mandarin dipping (214) tones may be associated with the most neck activity and with females dropping their head/chin. Further research will be vital to describe visual tone cues more precisely, in both perception and production. Shaw and colleagues have recently analyzed electromagnetic articulography (EMA) data on tongue blade and jaw movement in production across Mandarin tone–vowel combinations (Shaw, Chen, Proctor, Derrick, & Dakhoul 2014). Their results showed some differences in tongue blade position as a function of tone, and a physiologically mediated relationship between tongue blade and jaw position that differed as a function of both vowel color and tone identity. However, the perceptual salience of these tongue and jaw movements or of their relational invariance has yet to be determined. Given the common involvement of F0 in intonation and tone, the intonation and prosody literature may also be informative. Scarborough, Keating, Mattys, Cho, and Alwan (2009) reported that mouth opening movements (in particular, chin displacement) were most important for the visual perception of pitch-accented English syllables (involving F0, amplitude, and duration), with other rigid and nonrigid motion (head and eyebrow movements, respectively) making a small, independent contribution. Other research has also implicated head and/or eyebrow movements in prosody (Cavé et al., 1996; Cvejic, Kim, & Davis, 2010; Krahmer & Swerts, 2007; Munhall, Jones, Callan, Kuratate, & Vatikiotis-Bateson, 2004; Yehia, Kuratate, & Vatikiotis-Bateson, 2002), although the relationship with F0 does not appear to be straightforward or invariably evident (Ishi, Haas, Wilbers, Ishiguro, & Hagita 2007).

Consistent with the PAM’s findings with cross-language segmental influences, visual information may enhance cross-language AV speech perception (e.g., Hardison, 1999; Navarra & Soto-Faraco, 2005), but difficulty in using certain L2 visual cues has also been attributed to influence from an L1 (Wang, Behne, & Jiang, 2008). Ortega-Llebaria, Faulkner, and Hazan (2001), for instance, found that Spanish listeners failed to use visual cues that disambiguated contrasts that are phonemic in English but have allophonic status in Spanish—that is, visual cues that were disregarded in an L1 were not used in an L2, even when they could have been helpful. Thus, as with auditory speech perception, visual sensitivity to phonologically irrelevant contrasts may be attenuated, and new visual categories may need to be established by L2 learners (see also Pons, Lewkowicz, Soto-Faraco, & Sebastián-Gallées 2009).

A number of factors have been posited to affect the use of visual cues by nonnative perceivers (Hazan, Kim, & Chen, 2010). In applying the PAM to visual speech perception, the use of visual cues by nonnative perceivers would be expected to be most affected by the relationship between the inventories of visual cues in the L1 and L2. In a PAM-like notion, Hazan et al. (2006) pointed out that, similar to the case of auditory cues, there are three possible relationships between the inventories of visual cues in L1 and L2: (1) The same visual gesture may occur in both L1 and L2, marking the same phoneme distinctions; (2) a visual gesture in an L2 may have no counterpart in an L1; or (3) a visual gesture may occur in the L2, but mark different phoneme distinctions than in the L1. L2 categories may be novel in terms of their auditory distinctions, visual distinctions, or both, and differing levels of difficulty may be associated with each domain for a given novel L2 category (Wang, Behne, & Jiang, 2009).

### The present study

Although the PAM does not currently make specific reference to the use of visual cues to speech, the notion of visual information assisting speech perception is compatible with the PAM’s underlying viewpoint that speech perception involves the perception of amodal *articulatory* information (Best, 1995; Best & Tyler, 2007), in part due to the multimodal nature of natural speech and face-to-face communication. Despite the compatibility of the PAM and visual speech information, to our knowledge, little or no research has examined cross-language visual speech perception in light of this model, no research has investigated the PAM and Thai target tones, and most certainly, no research has applied PAM to the auditory–visual perception of lexical tones. So and Best (2010, 2011, 2014) have applied the PAM to auditory Mandarin target tones; Mandarin has four tones, which are maximally distinctive in tone space. With three level tones, Thai is a more complex tone language. The ability to cross-map tones to native intonation (English) and tone (Cantonese and Mandarin) categories is likely related to the nature of the target tone language. Here we investigated whether the PAM can be applied to Thai, or whether previous findings regarding the possible applicability of the PAM to tones were specific to Mandarin. We included two nonnative tone groups, Cantonese (three level tones, six in total) and Mandarin (one level tone, four in total), which have very different tone systems (see Fig. 1). Given that the perception of level tones in a foreign tone language may be directly (Qin & Mok 2011) or inversely (Chiao et al., 2011) affected by the number of level tones in the listener’s native tone language, these nonnative tone groups could be expected to have very different cross-mapping relationships to Thai (three level tones, five in total).

Although the link between tone and visual speech might seem to be an esoteric, low-incidence issue, in fact it is not. As we mentioned above, over 70 % of the world’s languages are lexical tone languages (Yip, 2002), over half of the world’s population speaks a tone language (Fromkin, 1978), and the research that we have just summarized indicates that the use of visual information in speech perception is ubiquitous. In order to accommodate the breadth of speech perception in theories, the range of L1–L2 interactions in L2 learning, and the range of cues that L2 learners use, additional information is required on the category assimilation of both lexical tone and visual speech information. Firstly here, in order to investigate the tone category assimilation issue, Cantonese and Mandarin speakers were asked to map Thai tones onto their own native lexical tone categories, and English speakers were asked to do the same, but with native intonation (prosodic) categories. Given the potential difficulty associated with this task for English speakers, as a control, a further group of English speakers were asked to complete the categorization task using simple symbols. Secondly, in order to investigate the visual speech issue, the tone stimuli were presented in AO, AV, and VO conditions. From these category assimilation data, predictions based on the PAM were then tested with discrimination data.

## Method

### Participants

- Thirty-six native-speaking Thai listeners (15 males, 21 females, mean age 29 year, *SD* =4 years) were recruited from the University of Technology, Sydney, and from other language centers in Sydney, Australia. Their mean duration of time spent in Australia prior to testing was 2 years (*SD* =2.8). Most had just arrived in Australia or self-reported that they were using Thai most of the time in daily life.
- Thirty-six native-speaking Mandarin listeners (11 males, 25 females, mean age = 25 years, *SD* = 3.7 years; mean duration spent in Australia prior to testing = 1 year, *SD* = 0.7) were recruited from the University of Western Sydney, University of Sydney, and University of Technology, all in Sydney, Australia. Most came from the People’s Republic of China, with two participants from Taiwan. Most had either just arrived in Australia or self-reported that they were predominantly using Mandarin or other Chinese dialects (excluding Cantonese) in their daily life.
- Thirty-six native-speaking Cantonese listeners (13 males, 23 females; mean age = 22 years, *SD* = 1.9 years; mean duration in Australia prior to testing = 1.5 years, *SD* = 2.2) were recruited from the University of Western Sydney, University of New South Wales, University of Technology, and other language centers in Sydney, Australia, or from the Chinese University of Hong Kong, Shatin, New Territories, Hong Kong. All of the participants recruited in Australia came from Hong Kong (*N* = 29), and most had either just arrived in Australia or self-reported that they were predominantly using Cantonese or other Chinese dialects in their daily life. Mandarin is part of the school curriculum in Hong Kong, so all participants had had some level of exposure to Mandarin; however, they were accepted into this study as long as they self-reported that they did not use Mandarin in their everyday life and their self-report score on their ability to use Mandarin was low.
- Thirty-six native-speaking Australian English listeners were recruited from the University of Western Sydney, Australia. Eight males and 28 females took part, and their average age was 24 years (*SD* = 7.3).

None of the participants had received any formal musical training longer than five consecutive years, for musical experience has been found to facilitate tone perception (Burnham, Brooker & Reid, 2014a). All participants were given a hearing test and had normal hearing (at or under 25 dB at each of 250, 500, 1000, 2000, 4000, and 8000 Hz). All of the non-Thai participants were naïve to the Thai language, and the English listeners were also naïve to any other lexical tone languages. All participants gave informed consent to participate in the experiment and received AUD$30, or an equivalent compensation for participation for the Cantonese participants in Hong Kong, or received course credit. The study was conducted under University of Western Sydney Human Research Ethics Committee approval.

### Stimulus materials

All of the participants completed two tasks, a category assimilation (or identification) task and a discrimination task. The stimulus materials for each are described in turn below. Thai language tones were used as stimuli in both tasks. It is worth noting that the number of tones in Thai (five) lies between the corresponding numbers for the other tone-language groups in the experiment, Mandarin (four) and Cantonese (six), so there could be no one-to-one correspondence between the set of Thai tones and the tone set for either Mandarin or Cantonese. Similarly, there was no obvious one-to-one correspondence between the Thai tones and the English intonation categories, given that English intonation patterns are realized across sentences rather than single syllables.

#### Category assimilation task

The category assimilation task stimuli consisted of one syllable spoken with each of the Thai five tones using a long vowel, /fū:/, /fù:/, /fû:/, /fú:/, /fǔ:/, of which only the first and third syllables are words in Thai (/fū:/ means “to rise/swell,” and /fû:/ means “the sound of blowing/hiss”). Only one syllable, /fu:/, was used in this experiment, and it was specifically chosen because it is the only syllable that can co-occur with all tones in Mandarin and Cantonese (i.e., that is, it is a meaningful word with each tone in both of these languages). This is important, since in this experiment we investigated how the native speakers of other tone languages would map nonnative tones onto the tones in their system, and it is impossible to write nonword characters in Traditional Chinese languages (though it *is* possible to do so in Thai orthography, and Thai text is used here to spell out both the words, mid and falling tones, and the nonwords, rising, low and high tones).

**Fig. 2:**
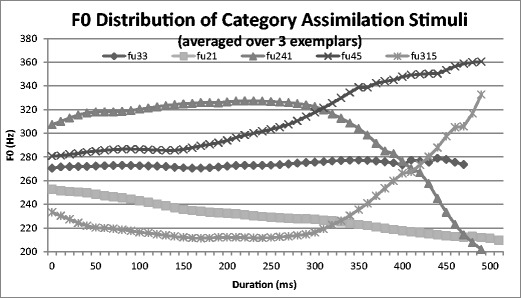
Fundamental frequency (F0) distribution of Thai tone stimuli in the category assimilation task, based on one female production of “fu:” (all produced with a long vowel), averaged over three exemplars for each of the Thai tones

The stimulus words were spoken by a 27-year-old native Thai female. The speaker was required to read aloud the syllables that were displayed on a screen. The productions were audiovisually recorded from a straight, face-on view in a sound-treated booth using a Lavalier AKG C417 PP microphone and an HDV Sony HVR-V1P video camera remotely controlled with Adobe Premiere software, which stored the digital audiovisual recordings on a separate computer (video at 25 frames/s and 720 × 576 pixels; audio at 48 kHz, 16-bit). Many repetitions were produced by the speaker, but only three exemplars of each syllable were selected for each tone. The original recordings were labeled using Praat (Boersma, 2001), and the corresponding videos were automatically cut from Praat TextGrids using a MATLAB script and Mencoder software and stored as separate video files. To ensure that the whole lip gesture of each syllable was shown in its entirety, 200 ms of the original recording was retained at the boundaries when each syllable video file was cut. The sound level was normalized, and all videos were compressed using the msmpeg4v2 codec. The F0 track for each tone (averaged over the three exemplars in the stimulus set) is presented in Fig. 2.

#### Discrimination task

The discrimination task stimuli consisted of six Thai syllables (voiceless unaspirated /ka:/, /ki:/, /ku:/; voiceless aspirated/k^h^a:/, /k^h^i:/, /k^h^u:/), each with each of the five Thai tones spoken by the same native Thai female, under the same conditions as for the category assimilation task stimuli. The resulting 30 syllables were either words (*N* = 21) or nonwords (*N* = 9).

### Procedure

All participants first completed the discrimination task (the easier and longer task), and then the category assimilation task. This constant order was maintained in order to minimize the effects on discrimination performance of having first explicitly categorized (assimilated to L1) the target stimuli. The category assimilation task and then the discrimination task are described below.

#### Category assimilation task

**Fig. 3:**
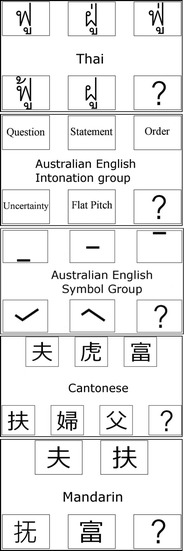
Category assimilation task response categories for each language group

Participants in all language groups completed a category assimilation task in which they were required to map the syllable that they heard to a corresponding same-tone word/nonword, character, symbol, or intonation type, depending on their native language group, as described below, choosing from a number of tone-different distractors. We note that for the native Thai comparison group, this was actually a straightforward identification task rather than a category assimilation (cross-mapping) task. English-speaking participants were divided into two groups of 18 participants, named the *intonation* group—testing the ability to map the stimulus syllable to the intonation system of Australian English—and the *symbol* group—testing the ability to map the stimulus syllable to a simple tone symbol (see Fig. 3).

For each of the language groups (a between-subjects factor), a 5 Tone × 3 Mode of Presentation within-subjects design was employed. The first within-subjects factor was *Thai Tone*; three exemplars of each of the five tone stimuli were presented in the test trials. The second within-subjects factor was *Mode of Presentation*: auditory only (AO), visual only (VO), or auditory–visual (AV). A further within-subjects factor was included in the experiment, that of the presence or absence of auditory background *Noise*: noise and clear. This was included as part of a larger study on visual speech (Burnham et al., 2014b), but since the influence of noise on the perceptual assimilation of nonnative phones has not been addressed in cross-language speech perception models, including PAM, only the clear-condition data were analyzed here.

The order of presentation of the AO, VO, and AV modes was randomized. In all, 180 randomized test trials were presented (consisting of AO/VO/AV presentations of three exemplars of the five Thai tones × 4 repetitions), split into four blocks. Two of the four blocks were with auditory noise, but the results of these trials are not considered here.

Participants were tested individually in a sound-attenuated room or a room with minimal noise interference on a Notebook Lenovo T500 computer running DMDX experimental software (see Forster & Forster, 2003). They were seated directly in front of the monitor at a distance of about 50 cm, and the auditory stimuli were presented via Sennheiser HD 25–1 II high-performance background-noise-canceling headphones, connected through the EDIROL/Cakewalk UA-25EX USB audio interface unit. Auditory stimuli were presented at a comfortable hearing level (60 dB, on average). The visual component of the stimuli (i.e., the face of the Thai speaker) was presented at the center of the computer screen in an 18-cm wide × 14.5-cm high frame. For the AO condition, a still image of the talker was shown. All participants were tested in a sound-attenuated booth using the same computer equipment, setup, and test room conditions.

Participants were instructed to use the mouse to click on the correct word/nonword, character, intonation type, or symbol (depending on their native language group) that was displayed on the screen. Figure 3 provides a summary of the response categories for each language group. Note that the response choices were arranged so that the left-to-right and right-to-left arrangements were counterbalanced between participants. Thai participants were asked to choose the Thai word/nonword with the correct spelling corresponding to the stimulus that they heard. English *intonation* participants were asked to choose an intonation type that the syllable they heard could fit into (question, statement, order, uncertainty, or flat pitch).^1^ Note that this group was informed that the word “order” in this context referred to someone giving a command. The English *symbol* participants were asked to choose a simple tone symbol that most fitted the pitch pattern of the syllable they heard. Mandarin participants were asked to choose from four simplified Chinese characters (representing the syllable /fu/ with all four Mandarin tones), and Cantonese participants were asked to choose from six traditional Chinese characters (representing the syllable /fu/ with all six Cantonese tones) that corresponded with the word that they heard. Thus, a participant only saw choices presented in their own language. In addition, the “unknown” category was given as a possible answer when participants were unable to select one of the provided keywords.

After their response on each trial, participants were asked to evaluate the “goodness” of the stimulus with respect to their chosen category, on a scale of 1–7 (“How good an example of this category is it?,” where 1 was a *very poor exemplar* and 7 was an *ideal exemplar*).

#### Discrimination task

The full details of the discrimination task are presented elsewhere as part of a larger study (Burnham et al., 2014b). Critical details are provided here.

A same–different AX discrimination task was employed. The within-subjects factor was the type of *Tone Pair*. Since Thai has three level tones and two contour tones (see Fig. 1), among the ten possible tone pairings were three level–level (LL) tone pairs, one contour–contour (CC) tone pair, and six level–contour (LC) tone pairs. Each of the ten possible tone pairs was presented four times in order to control order and same/different pairings; for a given pair of tone words, A and B, two *different* trials (AB, BA), and two *same* trials (AA, BB) trials were presented. All four trial types were presented twice with different exemplars. For each *same* trial, different tokens of the same syllable were used, so that the task involved a tone category match rather than an exact acoustic match.

There were three *modes of presentation*, AV, AO, and VO, and as for the category assimilation task, clear and auditory noise conditions were presented, but we report only the clear data here. Additionally, the *interstimulus interval* (ISI) was varied as a control factor, 500 or 1,500 ms (see Werker & Tees, 1984, for a description of possible phonetic and phonemic processing levels associated with these ISIs). In each language group, half of the participants were assigned the 500-ms and the other half to the 1,500-ms ISI condition. The initial consonant and vowel of the syllables (/ka:/, /ki:/, /ku:/; /k^h^a:/, /k^h^i:/, and /k^h^u:/) were also included as nested between-subjects factors (see Burnham et al., 2014b, for details), but these factors were not analyzed here and will not be mentioned further. Each participant received a total of 480 test trials (2 noise levels (noisy/clear) × 3 Modes AO/VO/AV × 10 Tone Pairs × 4 AB Conditions × 2 Repetitions). Noisy and clear trials were presented in separate blocks of 240 trials each, with only the clear stimuli being of interest here. The clear test file used here was split into two 120-trial test blocks, and each block combined 40 trials of each mode—AO, VO, and AV—presented randomly in order to avoid any attentional bias, with order counterbalanced between subjects. Within each 120-trial block, tone type, repetition type, and order were counterbalanced.

The test conditions and apparatus setup were as for the category assimilation task. Participants were instructed to listen to (AO, AV) and/or watch (AV, VO) a sequence of two videos of a speaker pronouncing syllables, and to determine whether the syllables were the same or different, pressing as quickly and accurately as possible the right shift key for “same” and the left shift key for “different.”

A discrimination index was calculated for each of the ten tone pairs in each condition, given by *dˈ* = *Z*(Hit rate) – *Z*(False positive rate), with appropriate adjustments made for probabilities of 0 and 1. *Hits* was defined as the number of correct responses (“different” responses on AB or BA trials). *False positives* was defined as the number of incorrect responses (“different” responses on AA or BB trials). According to signal detection theory, the *dˈ* measure is preferable for use in AX discrimination tasks, as opposed to raw percentages correct (see Macmillan & Creelman, 1991).

## Analysis and results

### Category assimilation task results

The top (most frequent) responses and associated percentages for the Cantonese tone, Mandarin tone, and English intonation groups (and the Thai native tone group for comparison) are included in the Appendix, along with mean goodness ratings. Also included in the Appendix are the top response percentages for the English symbol group, although this was a different type of categorization task for that group. The English symbol group achieved only 37.4 % accuracy in the AV condition and 35.9 % in the AO condition. In the VO condition, accuracy dropped to 24.3 %. In all conditions, the falling (241) tone was most commonly misidentified as mid (33), and high (45) was strongly misidentified as rising (315).

**Fig. 4:**
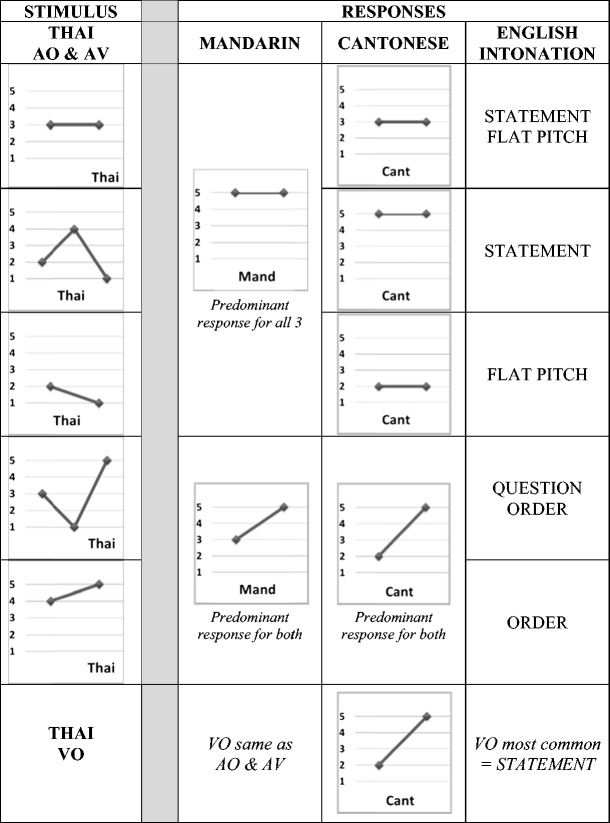
Summary of the predominant (>50%) category assimilation responses for the AV and AO (upper four rows) and VO (bottom row) conditions, with predominant responses based on the data in the Appendix. Note that, since no option was selected more than 50% of the time by the English Intonation group, assimilation patterns for the English intonation group are generally less robust than those for the Mandarin and Cantonese groups. Similarly, no VO option was >50% for any language group, and Cantonese responses to the Thai mid (33) and Thai low (21) tones were also <50%

A summary of the predominant assimilation patterns (based on the data in the Appendix) is shown schematically in Fig. 4. Note that we observed no strong assimilation patterns for the VO conditions in any group, or in any condition for the English intonation group (all <50 %).

Three separate multinomial logistic regressions were conducted on the categorization responses of the Mandarin, Cantonese, and the English intonation groups (since the focus here was on category assimilation, the English symbol group was not included in this analysis). To simplify the analyses, the tone inventories of both the stimulus and response languages were reduced to a few theoretically relevant categories. In the case of the Thai stimuli, the falling (241) and rising (315) tones were classed as the “falling” and “rising” stimuli, respectively, whereas the high (45), mid (33), and low (21) level tones were all classed as “level.” For the Mandarin tones, the falling tone (51) was classed as “falling”; the rising (35) and dipping (214) tones as “rising”; and the remaining tone, level (55), as “level.” In the case of the more complex inventory of Cantonese tones, the classification was as follows: low-falling (21) to “falling”; high-rising (25) and low-rising (23) to “rising”; and the remainder to “level”: high (55), mid (33), and low (22). Finally, the English intonation patterns were classed as follows: flat pitch to “level”; question to “rising”; statement and order to “falling”; and uncertainty to “other dynamic.”

The dependent measure was the categorization responses to the Thai tones by the various language speakers. Because this behavior was modeled using logistic regression, we predicted the log odds of the response being in one category relative to a baseline category. In the following analyses, the selected baseline category was the “level” category. The independent measures used were the tone class of the stimulus and its auditory–visual characteristics. In the case of independent measures with more than two levels, a baseline category was chosen: “level” for the tone features, and “auditory-only” (AO) for the auditory–visual features.

**Fig. 5:**
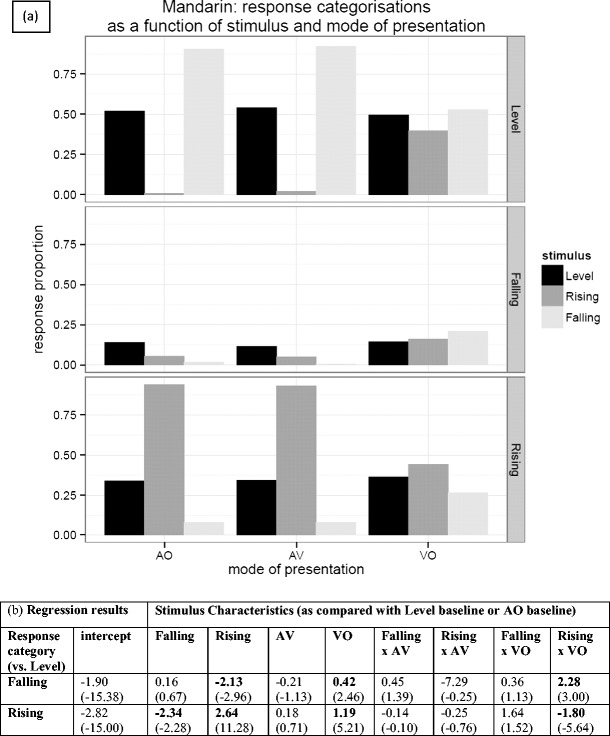
(**a**) Response categorizations for Mandarin speakers presented with Thai tones and asked to map them to their own tone inventory. Note that the bars represent the stimuli and the panels represent the participants’ responses. (**b**) Regression analysis results—the table entries are the coefficients from three multinomial logistic regression models, along with their associated *z* values in parentheses. Bold values indicate significant coefficients

Figure 5a gives the overall pattern of responses for Mandarin speakers mapping the Thai tone stimuli to their own tones. The most salient feature of Fig. 5a is the overall tendency of Mandarin speakers to treat all Thai tones as instances of the Mandarin level (55) tone, with a lesser tendency to treat the Thai rising tone as one of the two rising Mandarin tones. The Thai rising (315) tone was in fact predominantly assimilated to the Mandarin rising (35) tone (55 % in AV and 53 % in AO), although dipping (214) was also chosen a large percentage of the time (43 % in AV and 44 % in AO). Figure 5b gives the results from a multinomial logistic regression, and the model coefficients that are statistically significant support the veracity of these observations. If we take the coefficients relating to the falling stimulus characteristic for Mandarin in Fig. 5b (0.16 and −2.34), the tendency to classify the Thai falling tone stimulus as “falling” is not significant, but the tendency *not* to classify it as “rising” is significant. In contrast, we found a highly significant increase in the log odds of choosing “rising” over “level” if the participant was presented with a rising tone (2.64), and significant decrease in classifying it as “falling” (−2.13). In the case of the auditory–visual measures, it appears that augmentation of AO with vision had no significant differential impact on categorization between the baseline and other categories, whereas relying just on the visual stimulus (VO) tended to push respondents away from the “level” category toward both the “rising” and “falling” categories, though more strongly toward rising.

**Fig. 6:**
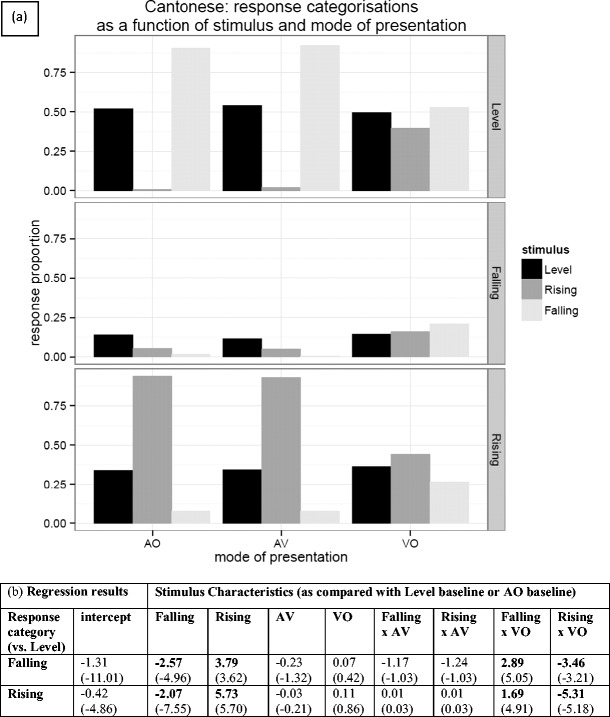
(**a**) Response categorizations for Cantonese speakers presented with Thai tones and asked to map them to their own tone inventory. (**b**) Regression analysis results—the table entries are the coefficients from three multinomial logistic regression models, along with their associated *z* values in parentheses. Bold values indicate significant coefficients.

The pattern for Cantonese respondents was a little different; Fig. 6a gives the overall pattern. There is a general tendency to map the Thai tones to either level or rising Cantonese tones, with falling Thai tones being treated as level Cantonese tones, and rising Thai tones treated as rising Cantonese tones. From the regression coefficients for Cantonese in Fig. 6b, we can see that a falling stimulus is overwhelmingly classified as “level,” with significant reductions in the log odds of both “falling” and “rising” classifications (−2.57 and −2.07, respectively). As can be seen from Fig. 6a, the vast majority of rising stimuli are classified as “rising.” For rising stimuli, we observed a significant move away from a “level” classification, even at the expense of classifying some rising stimuli as “falling.” Overlaid on this pattern of results was also a significant interaction with the VO condition, such that there was a significant reduction in the tendency to classify falling tones as “level,” and the exact reverse of this in the case of a rising stimulus.

**Fig. 7:**
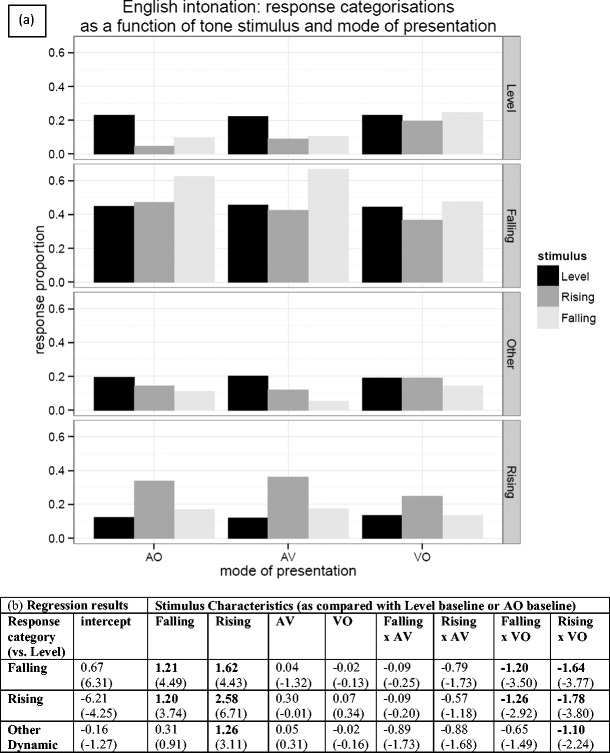
(**a**) Response categorizations for the English intonation group, presented with Thai tones and asked to map them to level (flat pitch), rising (question), falling (statement or order), or other dynamic (uncertainty) intonation patterns. Note that “falling” combines two falling categories, whereas all other categories incorporate only one response. (**b**) Regression analysis results—the table entries are the coefficients from three multinomial logistic regression models, along with their associated *z* values in parentheses. Bold values indicate significant coefficients

We found no strong assimilation patterns for the English intonation group; the incidence of each category being chosen was relatively low (all <50 %; see the Appendix). The overall pattern of the intonation group responses, shown in Fig. 7a, suggests that the “falling” category (statement or order) was favored on the whole (although note that “falling” is the only category that contains two intonation types), but there was a slight tendency for rising tones to be assimilated to rising intonation patterns (i.e., question). The results of the logistic regression in Fig. 7b show a significant increase in the log odds of *both* “falling” and “rising” classifications being associated with a falling stimulus, though with a bias toward falling. An analogous pattern of significance can be observed for the rising stimulus, but with a stronger bias toward a “rising” response (i.e., question). There is also a significant increase in the log odds of choosing “other dynamic” (uncertainty) over “level” if the participant is presented with a rising tone, but not with a falling tone. There is a significant overall decrease for both the “rising” and “falling” response categories in the VO condition, and both “rising” and “falling” classifications are significantly differentially affected by the VO condition, as evidenced by the significant VO × Rising and VO × Falling interactions. So, as for the tone languages, there is a shift away from the “rising” and “falling” categories (order, statement, and question), toward the “level” category (flat pitch) in the absence of audio input.

Taking the regression results as a whole, rising tones appear to have a privileged position over the other tones, in that there is a greater tendency to map rising Thai tones to an equivalent tone (and possibly intonation class) than for equivalent matchings in the other tone classes. For both the Cantonese and Mandarin groups, nonnative level and falling tones appear to be predominantly mapped to native level tones, whereas nonnative rising tones are mapped to native rising tones. The presence of both visual and auditory information did not appear to affect the relative classifications between the level baseline and other tone categories. Even within tone categories, the predominant assimilation patterns for the Mandarin and Cantonese groups were identical across the AV and AO conditions (see the Appendix). On the other hand, the absence of audio in the VO condition tended to push participants to spread their responses across more categories.

### Prediction of discriminability from the category assimilation task results

The category assimilation task results for the five Thai tones by the Mandarin, Cantonese, and English intonation groups were used to predict the discriminability of the ten tone contrasts used in the discrimination task, by classifying each of the ten tone pairs as one of the five “assimilation types” of the PAM: TC, UC, UU, CG, and SC. The  clearest order of predicted discriminabilities is TC > CG > SC (Best, 1995; Harnsberger, 2001); UC and UU are difficult to predict, since discrimination can range from poor, when they involve the use of the same exact assimilation categories, to moderate, for partially overlapping assimilation categories, to very good, for completely different assimilation categories (So & Best, 2014; see also Bohn, Avesani, Best, & Vayra, 2011, for caveats in predicting discriminability from UC and UU assimilation patterns).

For each of the five stimulus tones, if any one label was selected >50 % of the time (Best et al., 1996; Bundgaard-Nielsen, et. al. 2011; Bundgaard-Nielsen, et. al 2011b), it was classified as “categorized” (C); otherwise, it was classified as “uncategorized” (U). For the ten possible pairs of Thai tones (for each Language Group × Mode), if Tone X and Tone Y were both categorized and were categorized as different tones, then that pair was classed as TC (two-category assimilation). If Tone X and Tone Y were both categorized, but as the same tone, then this tone pair was classed as either SC or CG. In these cases, if the goodness ratings (see the Appendix) for Tone X and Tone Y did not differ significantly according to a *t* test, it was classed as SC; if they did differ significantly, it was classed as CG (see the following paragraphs for *t* test results). UC and UU classifications were assigned where appropriate, although these were not used in the analysis because it is difficult to make clear predictions (So & Best, 2014; see also Bohn et al., 2011).

For the VO conditions, there were no >50 % categorizations at all for any of the three category assimilation groups. As can be seen in the Appendix, across the AO and AV presentation modalities, Mandarin participants had more categorizations (10/10, 5/5 for both AO and AV) than did the Cantonese participants (6/10, 3/5 for both AO and AV). The Thai low (21) and mid (33) level tones were the tones uncategorized by Cantonese participants. The Thai group, unsurprisingly, made 10/10 correct categorizations. For the English intonation group, categorization was poor, since they made no correct categorizations at all in any condition.

The Mandarin and Cantonese were the only groups for which any CG/SC pairs were found (and then only in the AO and AV conditions), and the goodness ratings (on a scale from 1 to 7; see the Appendix) from the category assimilation experiment were analyzed using *t* tests on relevant CG/SC pairs to determine whether they were CG or SC pairs. Most *t* tests were nonsignificant, so SC labels were applied, but two *t* tests were significant, for the Mandarin group only—high versus rising in the AV (3.7 vs. 4.2) [*t*(289) = −1.97, *p* = .050] and AO (3.5 vs. 4.0) [*t*(300) = −2.16, *p* = .031] conditions—and so these two pairs were labeled CG (see Table 1). It was predicted that discrimination (as measured by *dˈ*) on TC tone pairs would be significantly higher than discrimination on CG and SC pairs, and that discrimination would be better on CG pairings than on SC pairings.

**Table 1:** PAM discrimination predictions and actual discrimination results (mean *dˈ*)

Predictions From Category Assimilation/ Categorization		Obtained *dˈ* Results in Discrimination				
		AV		AO		VO
	Thai Tone Pair		Mean *dˈ*		Mean *dˈ*	ALL UU
MANDARIN	21–33 *Low–Mid*	**SC**	3.5	**SC**	3.9	
	33–45 *Mid–High*	**TC**	3.6	**TC**	4.0	
	45–315 *High–Rising*	**CG**	3.9	**CG**	4.0	
	315–241*Rising–Falling*	**TC**	4.2	**TC**	4.1	
	21–241 *Low–Falling*	**SC**	3.7	**SC**	3.7	
	33–241 *Mid–Falling*	**SC**	1.3	**SC**	1.5	
	45–241 *High–Falling*	**TC**	3.9	**TC**	4.1	
	21–45 *Low–High*	**TC**	3.8	**TC**	3.6	
	21–315 *Low–Rising*	**TC**	3.9	**TC**	4.2	
	33–315 *Mid–Rising*	**TC**	4.4	**TC**	4.4	
	*Mean*	***TC***	*4.0*	***TC***	*4.1*	
		***SC&CG***	*3.1*	***SC&CG***	*3.3*	
		***SC***	*2.8*	***SC***	*3.0*	
		***CG***	*3.9*	***CG***	*4.0*	
CANTONESE	21–33 *Low–Mid*	UU	3.5	UU	3.2	ALL UU
	33–45 *Mid–High*	UC	3.5	UC	3.8	
	45–315 *High–Rising*	**SC**	3.7	**SC**	3.7	
	315–241*Rising–Falling*	**TC**	3.8	**TC**	4.0	
	21–241 *Low–Falling*	UC	3.6	UC	3.4	
	33–241 *Mid–Falling*	UC	2.2	UC	2.2	
	45–241 *High–Falling*	**TC**	4.0	**TC**	3.6	
	21–45 *Low–High*	UC	3.5	UC	3.5	
	21–315 *Low–Rising*	UC	3.3	UC	3.4	
	33–315 *Mid–Rising*	UC	3.9	UC	3.9	
	*Mean*	***TC***	*3.9*	***TC***	*3.8*	
		***SC***	*3.7*	***SC***	*3.7*	
ENGLISH SYMBOL	21–33 *Low–Mid*	UU	2.2	UU	3.4	ALL UU
	33–45 *Mid–High*	UC	3.4	UC	3.2	
	45–315 *High–Rising*	**SC**	3.5	**SC**	3.3	
	315–241 *Rising–Falling*	UC	3.4	UC	3.2	
	21–241 *Low–Falling*	UU	3.4	UU	3.7	
	33–241 *Mid–Falling*	UU	1.6	UU	1.8	
	45–241 *High–Falling*	UC	2.8	UC	3.1	
	21–45 *Low–High*	UC	3.4	UC	3.8	
	21–315 *Low–Rising*	UC	3.0	UC	2.8	
	33–315 *Mid–Rising*	UC	3.5	UC	3.5	
ENGLISH INTONATION		ALL UU				
THAI (for comparison)		ALL TC		ALL TC		ALL UU

A tone was considered categorized in the category assimilation experiment if any category corresponded to >50 %. TC = two-category, CG = category goodness, SC = single-category, UC = uncategorized–categorized and UU = uncategorized–uncategorized. Predicted discriminability in order from best to worst is: TC > CG > SC (these are in boldface), and the other assimilation pairs, UC and CC, are not analyzed here.

Although this was not an assimilation task for the English symbol group, PAM procedures were also applied to their data. Again, they produced no categorizations for VO. Using the 50 % criterion, they made only 4/10 categorizations for AO and AV, leading to one SC/CG pair in each mode. Applying *t* tests showed no significant difference in goodness ratings between high and rising for either AV (4.2 vs. 4.1) [*t*(163) = −0.52, *p* = .605] or AO (4.0 vs. 4.2) [*t*(154) = −0.88, *p* = .383], and hence these pairs were labeled SC. However, no discrimination predictions across pairs could be made, given that only one pair in each modality was labeled either TC, CG, or SC—in this case, SC. This is interesting in itself, since it shows that even with a more straightforward task (relative to the intonation cross-mapping), the PAM is not particularly useful in explaining tone discrimination patterns for the nontone English group.

### Discrimination task results

**Fig. 8:**
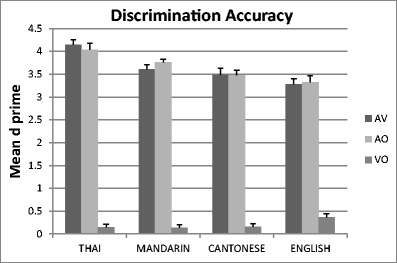
Discrimination accuracy results for each language group in each condition, averaged over tone contrasts (English results are pooled over the intonation and symbol groups)

Figure 8 presents the discrimination results for each language group averaged over tone contrasts. Table 1 summarizes the predictions and also gives the discrimination results by tone contrast for the Mandarin and Cantonese groups, as well as the English symbol group. It can be seen that assimilation patterns were identical across the AV and AO conditions. For the Mandarin group, a paired *t* test compared mean *dˈ*s across TC pairs to the mean *d’*s across SC/CG pairs. As predicted, TC pairs were discriminated significantly better than SC/CG pairs in both the AV [*t*(35) = 5.50, *p* < .001] and AO [*t*(35) = 5.95, *p* < .001] conditions. Furthermore, the CG pairing was discriminated significantly better than the SC pairings in both the AV [*t*(35) = −3.2, *p* = .003] and AO [*t*(35) = −4.9, *p* < .001] conditions. In contrast, for the Cantonese group, paired *t* tests comparing the TC and SC tone pairings showed no significant difference in either the AV [*t*(35) = 1.19, *p* = .242] or the AO [*t*(35) =0.38, *p* = .708] condition.

For the Mandarin group, then, the PAM predictions were upheld. It can be clearly seen that the mid–rising pairings that were best discriminated were indeed TC assimilation cases, and the mid–falling pairings that were worst discriminated were SC assimilations. For the Cantonese group, the best discrimination performance was indeed on a TC pair (high–falling).^2^ However, the single SC tone pairing (high–rising) was discriminated better by the Cantonese than was predicted by the PAM in both the AV and AO conditions. This finding of equivalence to TC pairs is the single deviation from PAM predictions.

Most of the UC and UU assimilation pairs were discriminated moderately well by the Cantonese group. However, as we suggested above, they varied widely (ranging from means of 2.2–3.9, with a significant difference between the highest and lowest means) [AV, *t*(35) = 6.13, *p* < .001; AO, *t*(35) = 5.73, *p* < .001]. Some UC cases were discriminated as well as TC cases (mid–rising), whereas others were relatively poorly discriminated (mid–falling). The latter pair was also discriminated particularly poorly by the English groups and by the Mandarin group, for whom it was an SC pattern.

Although no predictions could be made for the English intonation group, for whom all pairs were classed as UU, we found large variations in the UU pairs. In AV, the range of means for tone pairs was from 2.7 (for the pair mid–falling) to 4.1 (high–rising), with a significant difference between these lowest and highest pairs [*t*(17) = 4.73, *p* < .001]. In AO, the range of means was from 2.6 (mid–falling) to 3.9 (high–rising), with a significant difference between these lowest and highest pairs [*t*(17) = 4.72, *p* < .001]. For the VO condition (all classed as UU pairs for all language groups), we also observed significant differences among the maximum and minimum UU pairs: Thai, range of means from −0.17 (low–rising) to 0.58 (falling–rising), *t*(35) = −14.4, *p* < .001; Mandarin, range of −0.29 (mid–falling) to 0.63 (falling–rising), *t*(35) = −17.1, *p* < .001; Cantonese, range of −0.31 (high–falling) to 0.80 (mid–high), *t*(35) = −10.0, *p* < .001; English intonation, range of −0.22 (high–falling) to 1.15 (mid–rising), *t*(35) = 2.7, *p* = .015.

These significant differences demonstrate the large variations found for UC and UU pairs across the language groups, suggesting that accounts based on phonetic, and even acoustic, similarity and overlap between nonnative and native tones are likely to be required, in addition to those based on phonological categories (see the similar suggestion in So & Best, 2014).

## Discussion

Using Best’s (1995) perceptual assimilation model (PAM), we investigated the auditory–visual perception of Thai tones by native speakers of three tone languages (Thai, Cantonese, and Mandarin) and a nontone language (English). Application of the PAM predictions to Mandarin discrimination performance for Thai tones was successful: Discrimination was significantly higher for TC tone pairs than for the CG and SC pairs, and CG tone pairs were discriminated better than SC pairs. That the PAM predictions for the Mandarin group were borne out in the discrimination data provides some support for the applicability of PAM to lexical tones. However, the results for the Cantonese group were a less good fit with the PAM predictions; although the Cantonese participants discriminated their TC pairs relatively well, their single SC pair was discriminated equivalently well. PAM predictions based solely on the assimilation of nonnative contrasts to native phonological categories were unable to account fully for this specific discrimination result for the Cantonese group, suggesting that the PAM principles of predicting discrimination performance from cross-mapping assimilation patterns may not be fully generalizable to all tone-language pairings.

Discrimination predictions for the UC and UU cases were not analyzed here because it was difficult to make clear predictions (see Bohn et al., 2011, for caveats when predicting discriminability from UC and UU assimilation patterns), although clearly there was a wide range of discrimination performances between these categories. The particular difficulty in discrimination experienced by Cantonese participants with the UC pairing mid–falling (33–241) suggests that this pair differs in some way from the other UC pairs for Cantonese perceivers. The Mandarin perceivers’ difficulty with this same Thai contrast was predicted by the PAM, since they assimilated it as an SC pair. It is worth noting that the Thai falling (241) tone was assimilated by the Cantonese group to the high (55) tone, and that in both AV and AO, the second most common Cantonese response to the Thai mid (33) tone was also the high (55) tone (29 % and 27 %), which is still quite a large proportion. This category overlap may help explain the discrimination difficulty associated with the mid–falling (33–241) pairing (see Bohn et al., 2011). So and Best (2014) also found substantially different degrees of discrimination among UC pairings for nonnative perception of Mandarin tone contrasts, which they suggested was due to the listeners being substantially influenced by phonetic characteristics, not just by phonological categories (i.e., phonetic similarities between categories also play an important role).

Overall, Mandarin participants appeared to find the category assimilation task easier than did Cantonese participants (in terms of having a higher number of categorizations; i.e., tones were heard as a single native tone category), particularly for Thai level tones. We note that Mandarin participants had fewer native response categories (four vs. six) to choose from; given that the Cantonese tone space is more crowded, fine phonetic detail (e.g., the vocal quality/creak associated with the Cantonese low-falling [21] tone; Vance, 1977; Yu & Lam, 2011) may be more important for Cantonese perceivers when determining native tone category membership. For the Cantonese group, the Thai low (21) and mid (33) tones were difficult to categorize (<50 %), probably due to the fact that there are several options for a fairly level tone in both of those frequency regions. We note that for the Mandarin group, the Thai low (21) tone was categorized, but at levels of <57 %, which could have been due to the Mandarin inventory simply including no level-tone equivalent in that frequency region; Mandarin has only one *high* level tone. The Thai rising (315) tone also appeared to provide a challenge for the Mandarin participants in terms of choosing between Mandarin rising (35) and dipping (214) tones, although the former was the predominant category chosen (at <55 %).

The multinomial logistic regression analyses revealed that both the Mandarin and Cantonese groups generally showed a pattern of mapping Thai falling tones to native  level tones and Thai  rising tones to native  rising tones. That such consistency was found across languages suggests the possibility that something may be special about rising as opposed to falling tones. There may be a physical bias regarding sensitivity to F0 direction; Krishnan, Gandour, and Bidelman (2010) measured the frequency-following response (FFR) to Thai tones and found that over and above tone-language listeners (Thai and Mandarin) having more sensitive brainstem mechanisms for representing pitch (tracking accuracy and pitch strength) than do nontone (English) language perceivers, tone- and non-tone-language listeners can be identified by their degrees of response to rising (but not falling) pitches in the brainstem.

Although the Mandarin falling (51) tone is the most frequently used Mandarin tone (Wan, 2007), responses did not reflect this weighting, since falling (51) was in fact never the predominant response to any of the Thai tone stimuli. It is possible that durational cues played a role: Falling (51) is typically the shortest of the four Mandarin tones^3^ (Tseng, 1990), and our laboratory recordings of native Mandarin speakers have shown an average duration of 270 ms for the syllable /fu/ on falling (51). The Thai tone stimuli used here (which at around 500 ms were much longer than the Mandarin falling [51] tone, and not appreciably different from each other, since they were all the same [long] vowel length; see Fig. 2), may have been perceived to be too long to be a falling (51) tone. Additionally, larger pitch ranges are typically used for falling tones than for rising tones in Mandarin, which may influence the criteria used by Mandarin speakers to label stimuli as rising or falling (Bent, Bradlow, & Wright, 2006).

Given the possibly privileged status of rising F0 patterns, the slight rising tendency of the Thai high (45) tone may be important (see Fig. 4, which shows that the Thai high tone is predominantly assimilated to rising native tones by both the Cantonese and Mandarin groups). Indeed the findings of the regression analyses regarding rising tones may have been even stronger if the high (45) tone was classed as a rising rather than a level tone (as it is traditionally labeled). The slight falling nature of the Thai low (21) tone (which is also traditionally classed as a level tone) may not be as salient, given that it was predominantly mapped to native level rather than native falling tones. This was the case even to some extent for the Cantonese group, for whom the top response was their level (22) tone (albeit only at rates of 38 %–44 %), rather than their own low-falling (21) tone option (chosen at rates of 26 %–34 %). As we mentioned previously, vocal quality/creak is associated with the Cantonese low-falling tone (21; Vance, 1977; Yu & Lam, 2011), and it is possible that such fine phonetic detail is important for Cantonese perceivers who do not choose that tone in response to Thai low (21). One caveat with regard to the Cantonese data on mapping falling to level tones is that a less common high-falling (53) allotone exists for the Cantonese high (55) level tone (see L. K. H. So, 1996); this context may be important when it is considered that the Thai falling (241) tone was predominantly mapped to the Cantonese high (55) tone (at rates of around 75 %)—so perhaps falling was in fact being mapped to falling.

For the English intonation group, the incidence of each category being chosen was relatively low (all <50 %), and there was a slight tendency for rising tones to be assimilated to a question intonation. However, the regression analysis did not strongly support the latter tendency. In terms of PAM, there were no categorizations (no categories used >50 %) at all in any condition; that is, no tone was heard/seen as any one native intonation category. Given that categorization in this task was poor, no predictions from the PAM could be tested for the intonation group. Of course, weaker assimilation patterns than those for the Mandarin and Cantonese groups were expected, given that there is a larger discrepancy between a lexical tone and a prosodic system (which is generally realized over a sentence rather than over a single syllable, as in the stimuli here) than between two lexical tone systems. So and Best (2011) found similar results regarding the percentage of English categorizations for Mandarin tones—44 % identified a Mandarin falling (51) tone as a statement, and 42 % identified a Mandarin rising (35) tone as a question. So and Best (2014), in contrast, reported stronger assimilation patterns of English participants listening to Mandarin tones—for instance, 70 % identified the Mandarin falling (51) tone as a statement, and 52 % the Mandarin rising (35) tone as a question. Their results suggest that Mandarin tones may be easier to assimilate to the English prosodic system than are Thai tones. Nevertheless, it is of interest that So and Best (2014) did not find that TC Mandarin tone pairs were discriminated better than UC pairs; that is, although the researchers were able to make some predictions based on the PAM, these predictions were not entirely upheld.

The results for the English symbol group show that even with a more straightforward task (relative to the intonation cross-mapping), the PAM is not particularly useful in explaining discrimination patterns for the English group. Although the English intonation group faced a difficult task in terms of mapping lexical tones to a prosodic system, the English symbol group also found their task difficult. In the audio conditions, they achieved only around 37 % accuracy. Only two of the Thai tones—high (45) and rising (315)—were categorized at levels of over 50 %, and one of these, the Thai high tone, was strongly misidentified as the Thai rising tone at levels of around 80 %. Again, this is interesting in the context of the slight rising nature of the Thai high (45) tone, and given the parallel tendency of the Mandarin and Cantonese groups to map the Thai high tone to a rising tone in their native inventories.

With respect to visual speech, the results were not robust. The addition of visual information in the AV as compared with the AO condition had almost no effect on the response categories chosen. In the VO conditions, there were no categorizations at all for any of the category assimilation groups, and no predominant patterns emerged. Thus, no PAM predictions could be tested. The regression analyses showed that the absence of audio in the VO condition tended to push participants to spread their responses across more categories (i.e., closer to chance). It can be seen that even for the Thai group (who were not cross-mapping), the response percentages for any one tone category in VO conditions were relatively low (all well below 50 %), and discrimination was not in fact significantly different from chance (*dˈ* =0) for any one tone pairing. The effects of visual speech are often investigated via augmentation of perception in AV versus AO conditions, rather than by performance in VO conditions (Burnham et al., 2014b). However, comparisons of this nature with the present data showed no statistical interaction between the TC versus the SC/CG effects and the AV versus AO modes for either the Mandarin or the Cantonese group; that is, the effects were similar with and without visual information.

It should be noted that even if a more stringent categorization criterion was used in the prediction phase of the analysis, such as a label being selected 70 % rather than 50 % of the time (see Bundgaard-Nielsen, et. al. 2011), this would not have made a large difference to the number of categorizations in the VO condition, nor in any condition for the Cantonese group (see the Appendix). However, the Thai high–rising (45–315) tone combination would not, under this more stringent criterion, have been labeled SC for the Cantonese group; rather, it would be labeled UC, and this would in fact fit better with the PAM predictions. Additionally, in the discrimination test we used a same–different AX task here, but we note that Best (e.g., Best et al., 2001) argued that a two-alternative forced choice task (AXB) allows for measurement of sensitivity to smaller stimulus differences than may be easily assessed with AX tasks (Macmillan & Creelman, 1991, p. 134). A limitation of both tasks used here is that only one speaker was used to create the stimulus set; it is possible that the auditory and visual information here may have been peculiar due to particular speaker idiosyncrasies, rather than intrinsic phonetic categorical differences.

In summary, we investigated two main foci in this category assimilation study: the applicability of the PAM to (i) visual speech and (ii) lexical tone. With respect to visual speech, we found no evidence suggesting significant cross-mapping, and so PAM predictions could not be tested. In retrospect, although there is strong evidence for visual speech information regarding tone and for the use of such information by human perceivers (e.g., Burnhamet al., 2001a; Burnham et al., 2001b; Mixdorff, Charnvivit, and Burnham 2005a; Smith & Burnham, 2012), it may be that visual information for consonants and vowels is stronger than for tones, so the results for visual speech here may or may not generalize to applications of the PAM to visual information for consonants and vowels. Future tests of the PAM in a visual speech context for consonants and vowels would be a worthy pursuit, given that the PAM rests upon a model of speech perception in which articulatory events are the focus of speech perception (see, e.g., initial A➔V cross-language speech perception findings with infants: Best, Kroos, Gates, & Irwin 2014; Best, Kroos, & Irwin 2010, Best, Kroos, and Irwin 2011).

Turning to the lexical tone focus, the Mandarin audio (AO and AV) results provide proof that the PAM can be applied successfully to the perception of lexical tone in an unfamiliar L2 by speakers of a tone-language L1. This is in accord with the conclusions of So and Best (2014). Nevertheless, the results for the English intonation group and for the other tone group (Cantonese) are less promising. First, with respect to the intonation group, there was no evidence for cross-mapping of lexical tones to an L1 nontone language (English). Turning to the other category assimilation tone group, Cantonese, their results suggest that the principles found with Mandarin perceivers may not be fully generalizable to all tone-language pairings (in this case, Thai to Cantonese). Further category assimilation studies with a range of tone languages of differing natures—for example, African tone languages, which tend to involve grammatical determinants and whose tone inventories mostly comprise level rather than contour tones (Childs, 2003)—will assist in defining the limits of the applicability of the PAM to lexical tone contrasts. It may be useful in future studies to ask pitch-accent speakers (such as Swedish speakers) to use their pitch-accent categories to categorize tones from another language (as did So & Best, 2010, for Japanese listeners), in order to help define when the PAM can be applied to tones and when it cannot.

Successful application of the PAM to lexical tones and to visual speech is a worthwhile pursuit, in order to bring a hitherto powerful model of L1 and L2 speech perception to bear upon a wider range of speech phenomena. In the course of such a quest, the PAM itself may need to adapt somewhat in order to assimilate such nonsegmental and/or nonauditory features of speech. It is also possible that some hybrid model may be necessary for some of these other speech features. In this regard, like So and Best (2014), we found large differences in discrimination performance across the UC pairings, suggesting that accounts based on phonetic, and even acoustic, similarity and overlap between nonnative and native tones are likely to be required, in addition to those based on phonological categories.

## Appendix

**Table 2:** Category assimilation/categorization task top (most frequent) response patterns in clear background audio, with tones labeled with Chao values, percentages of choices, and mean goodness ratings (1–7) in the cells

Top Response %		THAI		MANDARIN		CANTONESE		ENGLISH INTONATION		ENGLISH SYMBOL		
		Top	*Goodness*	Top	*Goodness*	Top	*Goodness*	Top	*Goodness*	Top	*Goodness*	*Accurate Response*
**AV**	**21** **Low**	**Low**		**55**		**22**		**Flat Pitch**		**Low**		
		82.4	*5.5*	56.9	*3.9*	43.5	*3.9*	36.1	*5.0*	46.3	*3.7*	
	**33** **Mid**	**Mid**		**55**		**33**		**Flat Pitch**		**Mid**		
		99.1	*5.9*	87.0	*3.5*	40.7	*4.2*	29.6	*5.0*	44.4	*3.9*	
	**45** **High**	**High**		**35**		**25**		**Order**		**Rising**		***High***
		91.7	*5.9*	85.2	*3.7*	67.1	*4.0*	38.0	*5.1*	81.5	*4.2*	*9.3*
	**315** **Rising**	**Rising**		**35**		**25**		**Question**		**Rising**		
		88.0	*5.9*	54.6	*4.2*	81.5	*3.9*	33.3	*5.1*	72.2	*4.1*	
	**241** **Falling**	**Falling**		**55**		**55**		**Statement**		**Mid**		***Falling***
		81.0	*6.0*	75.5	*3.5*	74.5	*4.1*	45.4	*5.0*	40.7	*3.7*	*14.8*
**AO**	**21** **Low**	**Low**		**55**		**22**		**Flat Pitch**		**Low**		
		85.2	*5.5*	56.0	*3.8*	38.0	*3.6*	35.2	*4.6*	41.7	*3.7*	
	**33** **Mid**	**Mid**		**55**		**33**		**Statement**		**Mid**		
		98.6	*6.0*	90.3	*3.5*	39.8	*4.1*	41.7	*5.1*	37.0	*4.3*	
	**45** **High**	**High**		**35**		**25**		**Order**		**Rising**		***High***
		91.7	*5.9*	81.9	*3.5*	67.1	*4.0*	43.5	*5.3*	79.6	*4.0*	*15.7*
	**315** **Rising**	**Rising**		**35**		**25**		**Order**		**Rising**		
		83.8	*5.9*	52.8	*4.0*	84.7	*4.0*	38.9	*5.0*	64.8	*4.2*	
	**241** **Falling**	**Falling**		**55**		**55**		**Statement**		**Mid**		***Falling***
		85.6	*5.9*	78.7	*3.7*	74.5	*4.0*	39.8	*4.7*	35.2	*3.4*	*20.4*
**VO**	**21** **Low**	**Low**		**55**		**Unknown**		**Flat Pitch**		**Mid**		***Low***
		24.5	*3.8*	41.7	*3.9*	18.1	*–*	26.9	*3.3*	43.5	*3.9*	*25.9*
	**33** **Mid**	**Mid**		**55**		**25**		**Statement**		**Mid**		
		27.8	*3.8*	33.3	*3.9*	18.5	*4.0*	27.8	*4.4*	39.8	*3.8*	
	**45** **High**	**High**		**35**		**25**		**Statement**		**Rising**		***High***
		29.2	*4.1*	31.5	*3.9*	23.6	*4.2*	28.7	*4.6*	29.6	*4.0*	*6.5*
	**315** **Rising**	**Rising**		**35**		**25**		**Question**		**Rising**		
		30.6	*3.9*	32.4	*3.7*	22.2	*4.3*	25.0	*4.2*	31.5	*3.8*	
	**241** **Falling**	**Mid***		**55**		**Unknown**		**Statement**		**Mid**		***Falling***
		31.9	*3.6*	35.6	*3.9*	22.7	*–*	35.2	*4.3*	36.1	*4.2*	*17.6*

*Inaccurate Thai response

## Footnotes

1 A very simplified description of these intonation categories is as follows: In English, a question is often signified by a rising F0, and a statement by a falling F0. An order (command) might also be signified by a falling F0 (Wells, 2006), although this might be more abrupt than the fall in F0 for a statement. Flat pitch involves a level pitch without movement, a.k.a. “monotone.” Uncertainty can be signified by a falling and then rising contour (Ward & Hirschberg, 1985).

2 Note that all tone pairs referred to in this section are pairs of Thai tones, and that the pair composed of high and rising (high–rising), for example, is not to be confused with the Cantonese tone high-rising.

3 In Mandarin, the falling (51) tone is typically the shortest, and dipping (214) the longest (Tseng, 1990), and durational cues may contribute to Mandarin tone perception in some circumstances (Tseng, Massaro, & Cohen, 1986). Duration may be less useful in Cantonese, in which low-falling (21) is typically the shortest tone; Vance (1976) found that the difference in vowel length among Cantonese tones was too small to be used for tone identification. Vowel length (short vs. long) is phonologically relevant in Thai, meaning that duration is less useful as a subsidiary cue to tone identity.
